# Risk factor control in secondary prevention of cardiovascular disease: results from the multi-ethnic HELIUS study

**DOI:** 10.1007/s12471-017-0956-5

**Published:** 2017-02-08

**Authors:** M. Minneboo, S. Lachman, M. B. Snijder, J. T. Vehmeijer, H. T. Jørstad, R. J. G. Peters

**Affiliations:** 1 Department of Cardiology, Academic Medical Center, Amsterdam, The Netherlands; 2 Department of Public Health, Academic Medical Center, Amsterdam, The Netherlands

**Keywords:** Cardiovascular disease, Secondary prevention, Risk factors, Ethnicity, HELIUS study

## Abstract

**Objective:**

To evaluate the quality of contemporary secondary prevention of cardiovascular disease (CVD), and the differences between six ethnic groups in a large, observational cohort.

**Design:**

We included participants with a self-reported history of CVD from the HEalthy LIfe in an Urban Setting (HELIUS) study, which investigates inequalities in health between six ethnic groups living in Amsterdam, the Netherlands. We quantified the proportions of patients who were at the preventive treatment goal according to the guidelines of the European Society of Cardiology for six risk factors: hypertension, dyslipidaemia, smoking, overweight, physical inactivity and diabetes mellitus, and the use preventive medication.

**Results:**

Of 22,165 participants, 1163 (5%) reported a history of CVD. Mean age was 54 years. Overall, 69% had a systolic blood pressure of <140 mm Hg, and 42% had a low-density lipoprotein (LDL) cholesterol of <2.5 mmol/l. Non-smoking was found in 67%. Body mass index (BMI) <25 kg/m^2^ was found in 24%, and 54% reported adequate physical activity. The mean number of risk factors per patient was three (±1.1) out of six, and only 2% had all risk factors on target. Across the ethnic groups, non-smoking was more prevalent in the Ghanaian and Moroccan groups than in the Dutch (*p* < 0.001 and *p* = 0.001, respectively); BMI <25 kg/m^2^ and adequate physical activity were less prevalent among all ethnic minority groups compared with the Dutch group.

**Conclusion:**

We found large treatment gaps in secondary prevention of CVD. Ethnic differences in risk factors were found; however, strategies to improve overall risk factor management may be mandated before designing ethnic-specific strategies.

## Introduction

Individuals with established cardiovascular disease (CVD) have a high risk of mortality and recurrent morbidity [1–3]. Secondary prevention of CVD is effective in decreasing this risk, and has been shown to reduce healthcare costs, increase economic productivity and improve quality of life [4, 5].

The European Society of Cardiology (ESC) guideline on CVD prevention provides clear goals for primary and secondary CVD prevention [6]. The implementation of this guideline in daily practice has been evaluated by international surveys. The recent EUROASPIRE IV survey (2012–2013) found that the majority of 7998 patients with established CVD (hospital arm) do not achieve the lifestyles, risk factor levels, and therapeutic goals recommended in the 2012 ESC guideline [7]. However, the EUROASPIRE surveyed recruited patients from selected hospitals, and the findings on secondary prevention may differ in a population that includes more ethnic minority groups.

Ethnic minority groups often have a higher risk of CVD due to the influence of genetic, environmental, social and cultural factors [8, 9]. For example, in the UK, CVD mortality rates among South-Asian men are higher than those of the general population,[10] and hypertension is highly prevalent among adults of African origin in the UK compared with European adults [11]. Amsterdam, the Netherlands, is home to multiple ethnic minority groups, with approximately 35% of its citizens being first- or second-generation non-Western immigrants [12].

The aim of our study was to evaluate the quality of secondary prevention in individuals with CVD by quantifying the number of secondary prevention goals achieved and the prescription of secondary prevention medication, and subsequently to analyse the inequalities in secondary prevention between six ethnic groups.

## Methods

### Study population

The HEalthy LIfe in an Urban Setting (HELIUS) study is a large, multi-ethnic, population-based cohort study in Amsterdam, the Netherlands. The HELIUS study aims to investigate ethnic inequalities in health, focusing on three major causes of disease: cardiovascular disease, mental health, and infectious disease. The details of the study have been described elsewhere [13]. In brief, from January 2011 to November 2015, HELIUS included similar numbers of individuals (aged 18–70 years) from six ethnic groups: South-Asian Surinamese, African Surinamese, Ghanaian, Turkish, Moroccan and Dutch. Subjects were randomly sampled through the municipality register of Amsterdam, and stratified by ethnicity. Data were collected by questionnaire, physical examination and biological samples. The study protocols were approved by the Academic Medical Center Ethics Review Board, and all participants provided written informed consent.

**Fig. 1:**
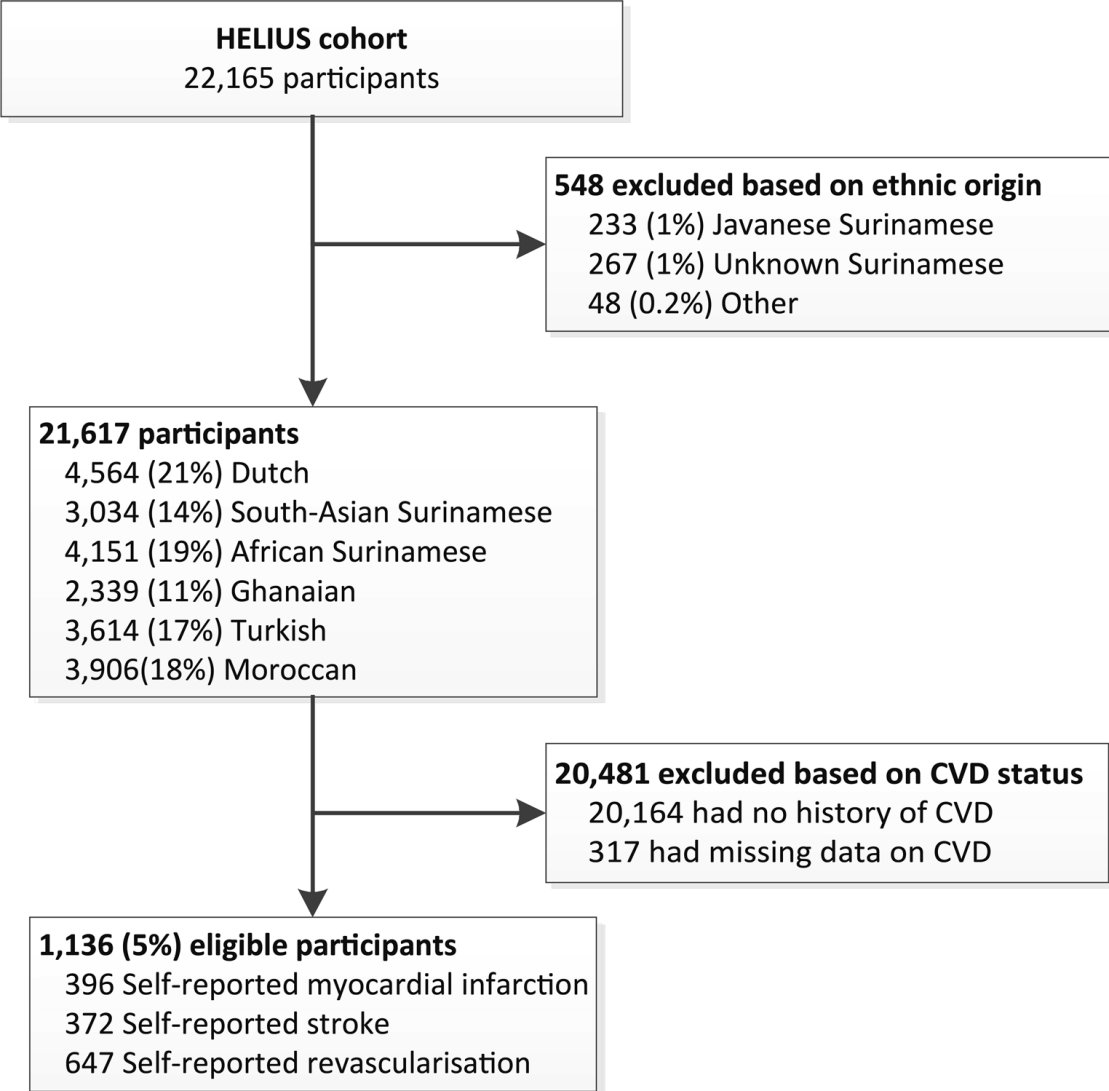
Flowchart: selection study population from HELIUS

In total, 22,165 participants were included for whom adequate data from the questionnaire and the physical examination were available. Due to very small numbers, participants with an unknown ethnic origin, unknown Surinamese origin or Java-Indonesian Surinamese were excluded. For the current analysis, we selected all participants who reported a history of CVD (‘patients’) (Fig. 1).

### Measurements

A history of CVD was based on three questions: 1) Have you ever had a heart attack? 2) Have you ever had a stroke? 3) Have you ever had a catheter intervention or bypass surgery on heart or legs? The third question was defined as revascularisation. Educational level was based on the highest qualification obtained in the Netherlands or abroad and was classified into two groups: 1) no or lower education (no schooling, vocational schooling or lower secondary schooling), 2) intermediate or higher education (intermediate vocational schooling, intermediate/higher secondary schooling, higher vocational schooling or university). Current smoking status was self-reported as yes or no. Physical activity was self-reported using the Short Questionnaire to Assess Health-Enhancing Physical Activity (SQUASH) questionnaire [14]. Adequate physical activity (yes/no) is defined as reaching the international goal for physical activity (moderate- to high-intensity physical activity for at least 30 min per day on at least five days per week).

Medication was identified and categorised using the Anatomical Therapeutic Chemical (ATC) classification system: blood pressure (BP) lowering medication, antithrombotics, lipid-lowering medication, and medication for diabetes mellitus. The BP was measured twice using a validated automated sphygmomanometer after five minutes of rest, and the mean of the two measurements was used. Height and weight were measured in light clothes without shoes. Body mass index (BMI) was calculated as weight (kg) divided by height squared (m^2^). Fasting blood samples were drawn to determine low-density lipoprotein (LDL) cholesterol, glucose and HbA1c.

Goals for secondary prevention were defined according to the 2012 ESC guidelines [6]: 1) systolic BP <140 mm Hg; 2) LDL cholesterol <1.8 mmol/l; 3) non-smoking; 4) BMI <25 kg/m^2^; 5) adequate physical activity 6) fasting glucose <7 mmol/l (patients without diabetes mellitus (DM)) or HbA1c < 48 mmol/mol (patients with DM). As the goal for LDL cholesterol was changed from <2.5 mmol/l to <1.8 mmol/l in 2012, we used <2.5 mmol/l for our main analysis, as the majority of the patients had experienced their events before 2012. Differences between ethnic groups were presented as prevalence ratios, with the Dutch group as the reference.

### Statistical methods

Continuous variables were presented as mean with standard deviation for normally distributed data, and as median with quartiles (Q1 and Q3) for non-normally distributed data. Categorical variables were presented as frequencies and percentages. Prevalence ratios and their corresponding 95% CI were calculated and graphs were created to display ethnic differences in the prevalence of achieved goals. Comparisons between groups were made by independent sample t‑tests, one-way ANOVA (and corrected for multiple testing using the Bonferroni method) and Fisher’s exact tests, as applicable. All statistical tests were two-tailed and a *p* value of <0.05 was used to indicate statistical significance. We used IBM SPSS Statistics, version 22.0 (IBM Corp., Armonk, NY, USA).

## Results

A total of 1136 patients, with a mean age of 54 years (±10.0) reported a history of CVD, including 276 (24%) South-Asian Surinamese, 237 (21%) Turkish, 221 (19%) African Surinamese, 167 (15%) Dutch, 130 (11%) Moroccan, and 105 (9%) Ghanaian patients. Of these, 501 (44%) were female. The median time from the CVD event to baseline examination was seven years (3–12 years). Most participants had had no education or only lower education (67%). The proportion of Dutch with intermediate or higher education was significantly greater compared with all the other ethnic groups (61% vs. ~28%, *p* < 0.001). Comparison of each ethnic group individually with the Dutch group also resulted in *p* values <0.001.

In total, 372 (33%) patients reported using the combination of BP-lowering, antithrombotic and lipid-lowering medication, and there were 371 (33%) patients who did not use any medication for secondary prevention of CVD. Dutch and South-Asian Surinamese had the highest use of medication while Ghanaians and Moroccans had the lowest use of medication (Table 1).

**Table 1:** Characteristics of CVD patients recruited from the HELIUS cohort

	Total		Dutch		South Asian		African		Ghanaian		Turkish		Moroccan	
					Surinamese		Surinamese							
	*n* = 1136		*n* = 167	(15%)	*n* = 276	(24%)	*n* = 221	(19%)	*n* = 105	(9%)	*n* = 237	(21%)	*n* = 130	(11%)
*Demographics*														
Age, years	54	(±10.0)	59	(±9.0)	56	(±9.7)	56	(±8.7)	52	(±8.2)	50	(±9.4)	49	(±11.1)
Age at CVD event, years	47	(±10.8)	52	(±10.1)	48	(±10.3)	48	(±10.9)	45	(±9.2)	43	(±9.6)	41	(±12.1)
Female	501	(44%)	63	(38%)	98	(36%)	107	(48%)	53	(50%)	116	(49%)	64	(49%)
*Education level*														
No or lower education	765	(67%)	65	(39%)	192	(70%)	146	(66%)	80	(76%)	188	(79%)	94	(72%)
Intermediate or higher education	371	(33%)	102	(61%)	84	(30%)	75	(34%)	25	(24%)	49	(21%)	36	(28%)
*Cardiovascular disease*														
Myocardial infarction	396	(35%)	58	(35%)	116	(42%)	64	(29%)	15	(14%)	110	(46%)	33	(25%)
Stroke	372	(33%)	66	(40%)	85	(31%)	108	(49%)	28	(27%)	51	(22%)	34	(26%)
Revascularisation	647	(57%)	93	(56%)	163	(59%)	91	(41%)	68	(65%)	151	(64%)	81	(62%)
*Medication use*														
BP-lowering medication	625	(55%)	109	(65%)	181	(66%)	135	(61%)	54	(51%)	110	(46%)	36	(28%)
Antithrombotics	548	(48%)	126	(75%)	169	(61%)	111	(50%)	19	(18%)	93	(39%)	30	(23%)
Lipid-lowering medication	553	(49%)	109	(65%)	175	(63%)	105	(48%)	32	(30%)	102	(43%)	30	(23%)
*History of diabetes mellitus*	321	(28%)	28	(17%)	128	(46%)	52	(24%)	25	(24%)	55	(23%)	33	(25%)
Diabetes medication	268	(83%)	22	(79%)	113	(88%)	46	(88%)	17	(68%)	46	(84%)	24	(73%)

*CVD* cardiovascular disease, *BP* blood pressure

Table 2 presents overall risk factor control according to goals as defined by the ESC [6]. Of all patients, 786 (69%) had a systolic BP <140 mm Hg and 470 (42%) patients had an LDL cholesterol <2.5 mmol/l, of whom 339 (72%) reported the use of lipid-lowering medication. There was a substantial variance in the different aspects of healthy behaviour: 67% reported non-smoking, 24% had a BMI <25 kg/m^2^, and an adequate level of physical activity was reported in 54%. In patients who did not report a history of DM and/or medication use for DM, we found 21 (3%) patients with fasting plasma glucose levels ≥7 mmol/l, i. e. not yet diagnosed diabetes.

**Table 2:** Prevalence of achieving CVD prevention goals according to the ESC guidelines 2007 and 2012

	Total population
*Blood pressure*	
SBP <140 mm Hg	786/1132 (69%)
SBP <140 mm Hg with medication	391/786 (50%)
SBP ≥140 mm Hg	346/1132 (31%)
SBP ≥140 mm Hg with medication	232/346 (67%)
*Cholesterol*	
LDL cholesterol <2.5 mmol/l	470/1112 (42%)
LDL cholesterol <2.5 mmol/l with medication	339/470 (72%)
LDL cholesterol ≥2.5 mmol/l	642/1112 (58%)
LDL cholesterol ≥2.5 mmol/l with medication	204/642 (32%)
LDL cholesterol <1.8 mmol/l	164/1112 (15%)
LDL cholesterol <1.8 mmol/l with medication	127/164 (77%)
LDL cholesterol ≥1.8 mmol/l	948/1112 (85%)
LDL cholesterol ≥1.8 mmol/l with medication	416/948 (44%)
Non smoking	755/1122 (67%)
Healthy weight (BMI <25 kg/m^2^)	270/1134 (24%)
Physical activity (≥30 min/5days/week)	613/1136 (54%)
*Diabetes mellitus regulation*	
Self-reported DM	321/1116 (29%)
Glucose-lowering medication	252/321 (79%)
HbA1c < 48 mmol/mol	105/315 (33%)
*Glucose regulation (in patients without self-reported DM)*	
Glucose <7 mmol/l	747/768 (97%)

*CVD* cardiovascular disease, *SBP* systolic blood pressure, *LDL* lower density lipoprotein, *BMI* body mass index, *DM* diabetes mellitus

**Fig. 2:**
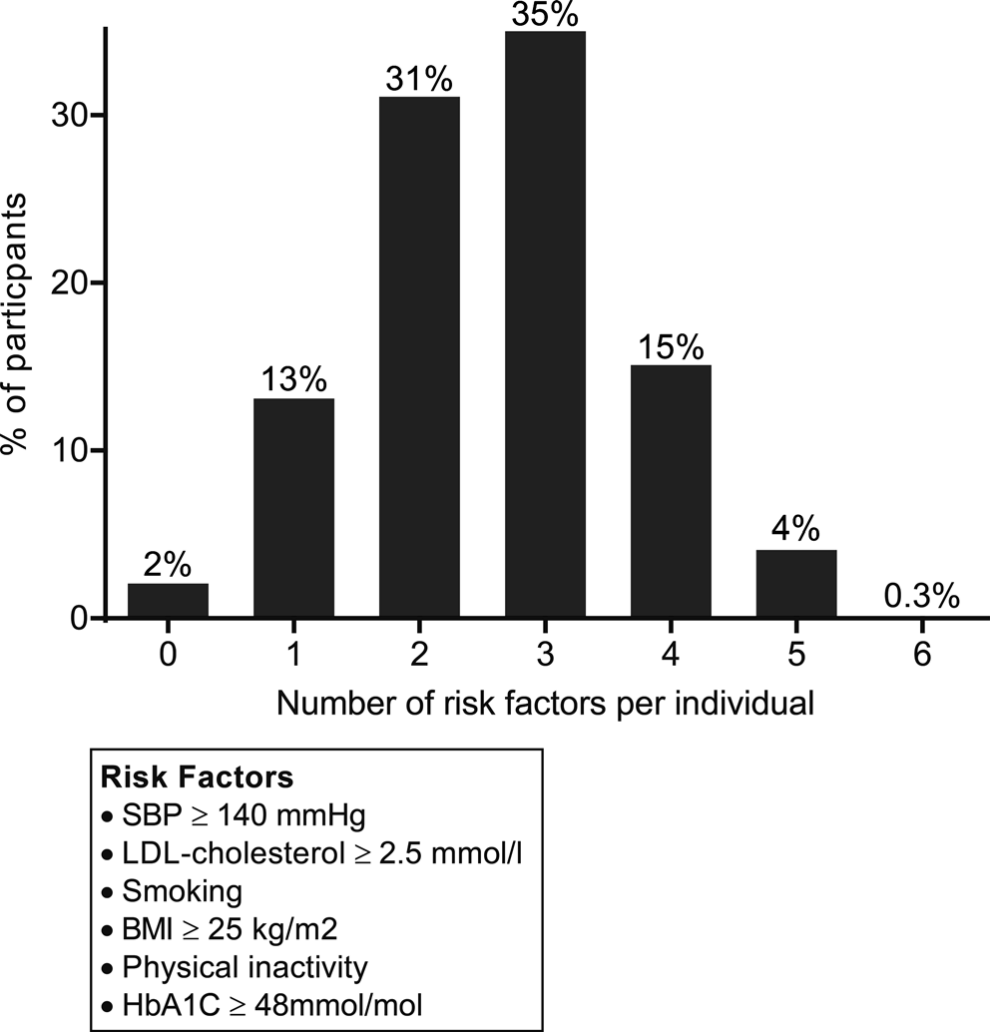
Distribution of number of risk factors off target per patient

The mean number of risk factors that were not on target per patient was 3 (±1.1) out of a maximum of six. This varied from 0 risk factors off target in 2% of the patients, to 4% of the patients with five risk factors off target (Fig. 2).

**Fig. 3:**
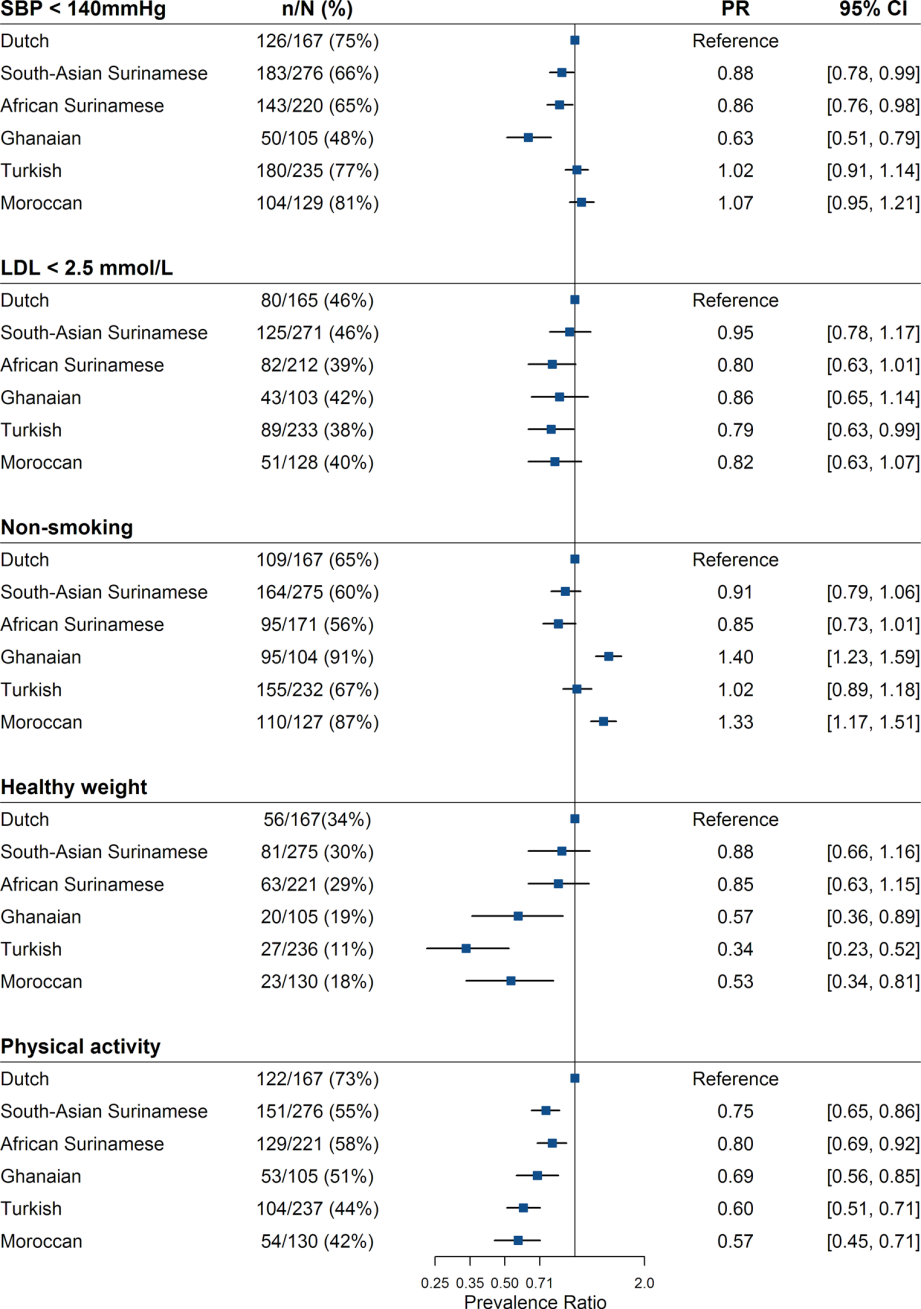
Prevalence ratios of achievement of secondary prevention goals among ethnic groups

Fig. 3 presents the prevalence ratios of cardiovascular goals per ethnic group. Systolic BP <140 mm Hg was most frequently observed in Moroccan, Turkish and Dutch patients (~76%), and least frequently in Ghanaian patients (48%). The proportion of patients with a systolic BP <140 mm Hg was significantly different between Ghanaians and Dutch (*p* < 0.001). The proportion of patients with an LDL cholesterol <2.5 mmol/l ranged from 38 to 46% across groups (*p* = 0.2 between groups). Non-smoking rates varied from 91% in Ghanaian (*p* < 0.001 vs. Dutch 65%) to 56% in African Surinamese patients (*p* = 0.8). A BMI of <25 kg/m^2^ was found in 11% of Turkish (*p* < 0.001 vs. Dutch 34%), 19% of Ghanaian (*p* = 0.09 vs. Dutch) and 28% of Moroccan patients (*p* = 0.02 vs. Dutch). Physical activity was most frequently on target in Dutch (73%), followed by African Surinamese (58%, *p* = 0.05), South-Asian Surinamese (55%, *p* = 0.002), Ghanaian (51%, *p* = 0.003), Turkish (44%, *p* < 0.001), and Moroccan patients (42%, *p* < 0.001). There were differences between ethnic groups in reported DM: Dutch patients had a low prevalence (17%) compared with South-Asian Surinamese (46%) patients (*p* < 0.001).

## Discussion

In our study, performed in individuals with a history of CVD in a large contemporary population-based cohort, we found that the proportion of risk factors on target for secondary prevention were disappointing, both for the total group and when stratified by ethnicity.

Overall, the mean number of risk factors that were not on target per patient was three (±1.1) out of six. The number of patients without any risk factors off target was strikingly low – only 2% (Fig. 2). There were substantial differences between ethnic groups in risk factor control. Non-smoking was most often seen in Ghanaians and Moroccans. Only a small proportion of Dutch were on target for healthy weight (34%), although most Dutch reported adequate physical activity levels (73%). This treatment gap in achieving lifestyle-related risk factors was even larger in all other ethnic groups.

We found that the use of BP-lowering medication, antithrombotics and lipid-lowering medication was less frequently reported in HELIUS when compared with a Dutch multicentre study. Only 55% of the patients used BP-lowering medication, 46% used antithrombotics, and 48% of the patients used lipid-lowering medication, as compared with 89% of the patients using beta-blockers, 92% acetylsalicylic acid (antithrombotics), and 93% lipid-lowering medications at the time of discharge in 2471 patients in the Netherlands [15]. These differences may be explained by the fact that HELIUS included participants from the general population with an average time since event of seven years, instead of selecting patients at hospital discharge. It is likely that our results give a more realistic impression of secondary prevention in daily practice.

When we compared our findings with the recent EUROASPIRE IV, both studies conclude that secondary prevention goals are not optimally met in clinical practice, although we did find some differences. First, smoking rates were lower in EUROASPIRE IV, with 84% non-smokers compared with 67% in HELIUS. However, smoking rates varied widely between ethnic groups and are likely to be associated with environmental, social and cultural factors [8]. Second, the goal of <2.5 mmol/l for LDL cholesterol was met in 44% of the patients in our study, while EUROASPIRE IV reported 58% of patients achieving this goal. Potential explanations include the much lower use of lipid-lowering medication in our study population, which may yet again be related to the difference in time elapsed since the event.

The HELIUS cohort consists of participants from six different ethnic groups. Our study shows a wide variation between these groups in the prevalence of risk factors and the use of secondary prevention drugs. Gijsberts et al. have previously reported ethnic-specific differences in a cohort consisting of Dutch, Chinese, Indians and Malays. They found significant ethnic differences in biomarkers for the severity of coronary artery disease [16]. In our study, Dutch patients were found to have better risk factor control and a higher prevalence of medication use. This could be related to socioeconomic status, which has been shown to be related to risk factor management and control in secondary prevention [17]. In HELIUS we found that the proportion of Dutch patients with an intermediate to higher education (a surrogate for economic status) is much greater than in the other ethnic groups, which could explain some of the differences between Dutch and other ethnic groups.

It has been suggested that ethnic-specific interventions are needed to optimise risk factor control in patients of different ethnic origin [18, 19]. However, control of risk factors in our study was poor overall, regardless of ethnicity. Based on our findings, strategies to improve overall risk factor management, or with special attention for patients with a lower social economic status, may be mandated before designing ethnicity-specific strategies.

### Strengths and limitations

There are several strengths to our study. First, we used a robust sampling technique from the source population through the municipality register of Amsterdam, stratified by ethnicity. This approach allows for a non-selective, community-recruited study sample as opposed to surveys that recruit their patients in selected hospitals or outpatient clinics, although we cannot exclude selection bias. Second, by design, HELIUS included ethnic minority groups, who are often underrepresented in epidemiological studies in high-income countries. Some limitations of our study require consideration. First, the HELIUS data are cross-sectional, and CVD risk factor changes over time could not be evaluated. Second, the diagnosis of CVD was self-reported, potentially leading to either under-reporting or over-reporting. For example, the overall percentage of women in HELIUS is relatively high (44%), especially in African Surinamese, Ghanaian, Turkish and Moroccan patients. Possibly, these participants may have over-reported having a diagnosis of CVD. However, previous studies have shown a high degree of specificity for self-reported CVD and stroke [20, 21]. Third, participants older than 70 years were not included in HELIUS, potentially resulting in a lower proportion of individuals with CVD, as the prevalence of CVD increases with age. Fourth, in our current analyses we did not adjust for the difference in educational level. Socioeconomic status likely impacts on adherence to preventive treatments in all ethnic groups. However, we believe that even if a significant proportion of the ethnic differences were associated with socioeconomic class, the message for daily practice would be unchanged, i. e. that the prevalence of insufficient secondary prevention is highest among ethnic minority groups and that they may require more intensive support. Finally, HELIUS included equivalent numbers of participants from each ethnic group; therefore, the overall findings may not be similar to those of a random sample from the same overall population.

### Conclusion

We found large treatment gaps regarding risk factor control in secondary prevention in the majority of individuals with CVD, with significant differences between ethnic groups. Overall, the use of secondary preventive medication is low, and markedly lower than reported in recent European surveys. Almost no patients (2%) have optimal risk factor control, with a mean of three out of six risk factors off target. While there is variation in the prevalence of risk factors and the use of prevention medication between ethnic groups, new initiatives should probably first aim to increase the overall quality of secondary prevention, regardless of ethnicity.
